# Photosynthetic and defensive responses of two Mediterranean oaks to insect leaf herbivory

**DOI:** 10.1093/treephys/tpac067

**Published:** 2022-06-29

**Authors:** Nikolaos M Fyllas, Despina Chrysafi, Dimitrios N Avtzis, Xoaquín Moreira

**Affiliations:** Biodiversity Conservation Lab, Department of Environment, University of the Aegean, Mytilene 81100, Greece; Biodiversity Conservation Lab, Department of Environment, University of the Aegean, Mytilene 81100, Greece; Forest Research Institute, Hellenic Agricultural Organization, Thessaloniki 57006, Greece; Misión Biológica de Galicia (MBG-CSIC), Apartado de Correos 28, Pontevedra, Galicia 36080, Spain

**Keywords:** chemical defenses, light response curves, *Lymantria dispar*, physical defenses, *Quercus coccifera*, *Quercus pubescens*

## Abstract

Insect herbivory is a dominant interaction across virtually all ecosystems globally and has dramatic effects on plant function such as reduced photosynthesis activity and increased levels of defenses. However, most previous work assessing the link between insect herbivory, photosynthesis and plant defenses has been performed on cultivated model plant species, neglecting a full understanding of patterns in natural systems. In this study, we performed a field experiment to investigate the effects of herbivory by a generalist foliar feeding insect (*Lymantria dispar*) and leaf mechanical damage on multiple leaf traits associated with defense against herbivory and photosynthesis activity on two sympatric oak species with contrasting leaf habit (the evergreen *Quercus coccifera* L. and the deciduous *Quercus pubescens* Willd). Our results showed that, although herbivory treatments and oak species did not strongly affect photosynthesis and dark respiration, these two factors exerted interactive effects. Insect herbivory and mechanical damage (vs control) decreased photosynthesis activity for *Q. coccifera* but not for *Q. pubescens*. Insect herbivory and mechanical damage tended to increase chemical (increased flavonoid and lignin concentration) defenses, but these effects were stronger for *Q. pubescens*. Overall, this study shows that two congeneric oak species with contrasting leaf habit differ in their photosynthetic and defensive responses to insect herbivory. While the evergreen oak species followed a more conservative strategy (reduced photosynthesis and higher physical defenses), the deciduous oak species followed a more acquisitive strategy (maintained photosynthesis and higher chemical defenses).

## Introduction

The antagonism between phytophagous insects and their host plants represents one of most ancient and widespread interactions on the planet (Labandeira 2007). Plants and insect herbivores together account for more than half of the described species worldwide (Cyr and Face 1993), and insect herbivory is thought to be a major driver of biodiversity (Coley and Kursar 2014) and ecosystem function (Huntly 1991). On one hand, the co-evolutionary dynamics between insect herbivores and plants are thought to have triggered accelerated rates of speciation in both groups (Futuyma and Agrawal 2009). On the other hand, phytophagous insects are estimated to consume close to a fifth of all plant biomass produced annually in natural ecosystems (Turcotte et al. 2014), representing a key factor controlling primary productivity and biomass turnover.

Much of the ecological research on plant–insect herbivore interactions has focused on understanding the influence of ‘bottom-up’ (resources and plant traits) and ‘top-down’ (predators and disease) factors on insect herbivory. Research focusing on bottom-up controls has long recognized that variation in plant nutrients or defensive traits plays a key role in shaping insect abundance and herbivory (Rhoades 1979, Agrawal 2011). These defensive traits can be constitutively expressed by plants (i.e., always present in the plant) or can be induced (i.e., only synthesized or activated in response to herbivore damage). In particular, plants respond to insect attack by producing diverse types of defensive traits, including chemical compounds (e.g., phenolics and terpenoids), physical structures (e.g., trichomes, thorns and toughed leaves) and phenological mechanisms (e.g., synchronous leaf flushes or fruit output to satiate herbivores) which reduce herbivore impacts on plant fitness (Agrawal 2011). These defensive traits, independently or concurrently, exert strong controls on the amount, pattern and timing of damage by insects on individual plants and are therefore a key factor shaping the outcome of plant–insect herbivore interactions and in turn ecosystem functions contingent upon herbivory.

Insect herbivory can also lead to induced changes in plant primary metabolism. Such changes include shifts in photosynthetic efficiency, growth rate and carbon and nitrogen remobilization (Zhou et al. 2015). Most experimental studies have reported a reduction of leaf photosynthesis after insect damage (Warrington et al. 1989, Bilgin et al. 2010, Visakorpi et al. 2018), although the reverse pattern has also been observed (Trumble et al. 1993, Retuerto et al. 2004). The downregulation of photosynthesis following insect herbivory has been attributed to increased energy demand to produce defensive metabolites, to infested leaf senescence and to the role of jasmonic acid as a signal for protection against oxidative damage (Nabity et al. 2013, Zhou et al. 2015). By contrast, stimulation of photosynthesis following insect herbivory has been commonly considered as a consequence of increased requirements for the production of defense metabolites to compensate for leaf area loss or the manipulation of plant metabolism by herbivores for their own benefit (Zhou et al. 2015).

Despite extensive research efforts, most previous studies assessing the link between insect herbivory, photosynthesis and plant defenses have been performed on cultivated model plant species (e.g., Brassicaceae, Solanaceae; Hall and Ferree 1976, Zhou et al. 2015), neglecting a full understanding of patterns in natural systems (but, see Copolovici et al. (2017) and Visakorpi et al. (2018)). A recent study has shown that leaf herbivory by the winter moth (*Operophtera brumata*) on pedunculate oak (*Quercus robur*) led to a 48% reduction in the potential photosynthesis and a 53% increase in the emission rate of isoprene (a signaling compound altering resistance to biotic and abiotic stresses) (Visakorpi et al. 2018). Here, we go a step further and investigate the effects of herbivory by a generalist defoliator insect (*Lymantria dispar)* (i.e., real herbivory) and leaf mechanical damage (i.e., simulated herbivory) on multiple leaf traits associated with defense against herbivory and photosynthesis activity on two sympatric oak species with different leaf economic strategies (the evergreen *Quercus coccifera* and the deciduous *Quercus pubescens*). Specifically, we asked: (i) Do photosynthesis-related traits and/or defenses of oak leaves change following leaf damage? (ii) Are the effects different between herbivore-induced damage versus mechanical wounding? (iii) Are the effects different between oak species? To address these questions, we quantified structural (size and toughness) and chemical (phenolic compounds) defensive traits as well as gas exchange-related traits (light saturation, photosynthesis and respiration rates) in leaves of both oak species. Overall, by measuring a suite of traits associated with primary and secondary metabolism, we build toward a more complete understanding of herbivore-induced plant responses in long-lived tree species.

## Materials and methods

### Study site and natural history

The study was carried out at the fields surrounding the University of the Aegean campus on Lesvos (Lat = 39.08, Long = 26.56) during the spring and summer of 2019. The main woody species found in the area are *Pinus brutia*, *Olea europaea*, *Q. coccifera*, *Q. pubescens* and *Pistacia lentiscus*. We selected 10 *Q. coccifera* shrubs and 10 *Q. pubescens* small trees (height <3 m) to implement our experimental set-up.

*Quercus coccifera* is a typical evergreen sclerophyllous shrub or tree that can withstand long and intense drought periods during Mediterranean summers due to its ability to tolerate very low soil water potentials by regulating its water loss (Vilagrosa et al. 2010). Leaves of this oak species flush mostly during spring (Karageorgou and Manetas 2006) and remain in the tree for up to 18 months (Diamantoglou and Mitrakos 1981). *Quercus pubescens* is a Mediterranean deciduous or semi-deciduous broadleaved oak that can withstand summer drought and maintains a relatively high leaf water content by compensating water loss with water uptake through an efficient hydraulic architecture (Vodnik et al. 2019). Leaves of this oak species flush in spring and remain in the tree from 7 to 8 months.

One of the common insects attacking both oak species is the gypsy moth *L. dispar* (Lepidoptera, Lymantriidae) (Southwood 1961, Damestoy et al. 2019). The natural distribution of gypsy moth expands from Western Europe and the Mediterranean basin to Central Asia (Paini et al. 2018). Caterpillars feed on leaves of >500 host plant species of different families, including both conifers and broadleaves (Liebhold et al. 1995, Damestoy et al. 2019). Outbreaks of gypsy moth can have significant economic and environmental impacts to forest and agricultural ecosystems (Alalouni et al. 2013). The phenology of *L. dispar* (egg hatching and larvae development) is well synchronized with oak phenology (bud bursting) and its larvae can cause extensive defoliation in spring and early summer (Damestoy et al. 2019). In addition, later instars can rapidly consume leaf area and bite through tough tissues, including secondary and primary leaf veins, while they significantly reduce tree photosynthetic rates (Copolovici et al. 2017).

### Experimental set-up

In April 2019, we collected first-instar gypsy moth caterpillars (1 week after egg hatching) from *Q. coccifera* and maintained them in large glass pots. In May 2019, we identified nine sunlit shoots with only intact leaves from each study tree and enclosed them separately in small mesh bags (with a mesh size <1 mm). Following the experimental design of Visakorpi et al. (2018), we randomly assigned each bag to one of the following induction treatments: (i) herbivory addition, (ii) mechanical damage or (iii) control so that each tree had three bags of each treatment. In each of the herbivory addition bags, we added two forth-instar gypsy moth caterpillars and allowed them to feed on the leaves for 5 days. After 5 days, we paired each herbivory addition shoot with a mechanical damage shoot and imitated the damage made to the leaf by the caterpillars (in herbivory addition treatment) by tearing the leaves in the mechanical damage treatment. Control shoots were left intact. We left the mesh bags around the shoots to prevent additional herbivory until the end of the experiment.

### Measurements of gas exchange traits

**Figure 1.**
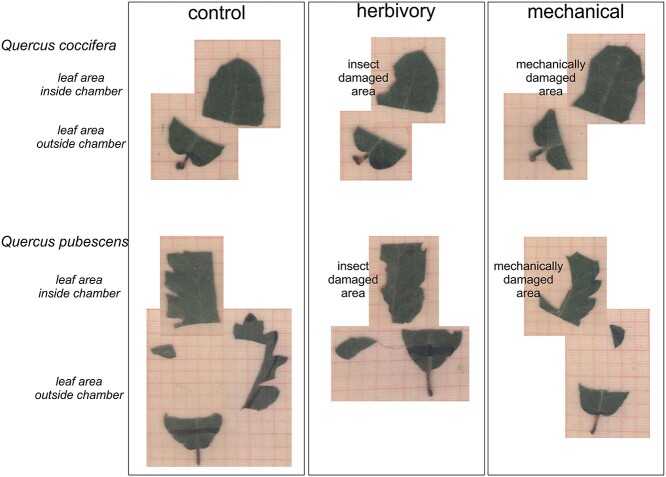
Description of leaf area measurements made across the three induction treatments and the two study species. **‘**Leaf area inside chamber’ was estimated in order to correct the CO_2_ fluxes, as in some leaves, the whole chamber area (6 cm^2^) was not fully covered either due to smaller leaf size or inclusion of ‘damaged area’ from induction treatments.

Fifteen days after establishing induction treatments, we randomly chose one fully sunlit leaf from each branch for each of the three treatments per plant (1 leaf × 3 branches × 3 treatments × 10 trees per species) for gas exchange measurements. We estimated photosynthetic light response curves for each leaf, assuming that within branch variation in gas exchange is considerably smaller compared with between branches and individual tree variation. For both control and damaged leaves (herbivory and mechanical damage), the whole leaf area was inserted in the chamber. The net photosynthetic rate per area (*A*_net_—μmol m^2^ s^−1^) was subsequently estimated after measuring the area of each leaf in the chamber and correcting the flux per unit area (Figure 1). We note that, in contrast to Visakorpi et al. (2018), where the photosynthetic rate of an intact part of the damaged leaves was measured, in our case, we used the whole leaf (including damaged areas) and subsequently corrected *A*_net_ for the leaf area that was actively involved in leaf gas exchange. The light response (*A* − *I*) curves were developed by making three logs at each one of the 16 light levels (2000, 1800, 1600, 1400, 1200, 1000, 800, 600, 400, 200, 100, 80, 60, 40, 20, 0 μmol m^2^ s^−1^) of photosynthetically active radiation (PAR) using the LICOR 6400 infrared gas analyzer (Licor Biosciences, Lincoln, NE, USA). We let the leaf settle to each new PAR level for at least 2 min before taking three logs. Relative humidity of the chamber was kept between 50 and 60%, temperature near 25 °C and the flow rate at 500 ml min^−1^. However, in some of the curves, stomatal conductivity substantially dropped during the measurements and these curves were excluded from our analysis. In total we had 21, 30 and 30 *A* − *I* curves for *Q. coccifera* and 17, 15 and 16 for *Q. pubescens* control, herbivory and mechanical damage treatment, respectively. Data from the light curves were used to estimate the *A* − *I* parameters (*A*_sat,lc_, *K_m_* and *R*_dark,lc_) as well the mean light saturated photosynthetic rate (*A*_sat_) by averaging all measurements above 1600 PAR for each leaf (nine logs) (Table 1).

**Table 1:** List of measured gas exchange-related and defensive traits, and their abbreviations, units and type of estimation. The proportional damage to leaf area (*E*_LA_) was estimated by the ratio of the damaged to intact *L_A_*.

Plant trait	Abbreviation	Unit	Estimation
Leaf area	*L_A_*	cm^2^	Measurements
Leaf thickness	*L*_th_	cm	Measurements
Leaf dry matter content	LDMC	g g^−1^	Measurements
Leaf dry mass per area	LMA	mg m^−2^	Measurements
Photosynthetic rate (light saturated)	*A*_sat,lc_	μmol m^−2^ s^−1^	Inferred from light response curve
Half saturation coefficient	*K_m_*	μmol m^−2^ s^−1^	Inferred from light response curve
Respiration rate (dark)	*R*_dark,lc_	μmol m^−2^ s^−1^	Inferred from light response curve
Photosynthetic rate (average)	*A*_sat_	μmol m^−2^ s^−1^	Measurements (average of nine logs)
Respiration rate (dark)	*R*_dark_	μmol m^−2^ s^−1^	Measurements (average of five logs)
Flavonoid concentration	Flavs	mg g^−1^	Measurements
Lignins concentration	Lignins	mg g^−1^	Measurements
Condensed tannins concentration	CTannins	mg g^−1^	Measurements
Hydrolysable tannins concentration	HTannins	mg g^−1^	Measurements

A separate leaf from the same branch was covered with foil and left in the dark for at least 5 min to mimic dark conditions and measure leaf dark respiration (*R*_dark_—μmol s^−1^ m^−2^). The leaf was then transferred into the chamber with no light and the CO_2_ exchange rate was monitored until there was no consistent change (around 30 min for *Q. pubescens* and around 20 min for *Q. coccifera*) and with a stable conductivity (usually >0.05 mol s^−1^ m^−2^). At this point, five logs at a constant CO_2_ atmospheric concentration of 400 p.p.m. were made for each leaf.

### Measurements of leaf traits

At the end of the gas exchange measurements, we collected leaves that were inserted in the chamber in addition to 10 leaves from the same branch to measure a set of structural and defense traits (Table 1). Chamber leaves and one leaf of similar size per branch were transferred to the laboratory and were placed in the fridge inside a bag with wet paper. One day later, we measured their wet mass (g) and thickness (*L*_th_—mm) for leaves. Using a portable scanner (iScan 900dpi), we scanned each leaf that was inserted in the chamber (all three treatments), and for the herbivory and the mechanical damage treatments, the additional intact leaf. In these images, we estimated the leaf area (*L_A_*—mm^2^) using the ImageJ image analysis software (Schneider et al. 2012). The proportional damage to leaf area (*E*_LA_) by each induction treatment was estimated by the ratio of the damaged to intact *L_A_*. We then oven-dried the leaves for 48 h at 60 °C and weighted them. We estimated leaf mass per area by dividing leaf dry mass to its area (LMA—g m^−2^) and leaf dry matter content by dividing dry to wet leaf mass (LDMC—g g^−1^). Leaf thickness, LMA and LDMC are correlated with leaf toughness and can therefore act as a proxy of structural defense against herbivory (Lill et al. 2006, Moreira et al. 2020).

The rest of the leaves per tree and treatment were aggregated to measure phenolic compounds as putative chemical defenses (Table 1). These compounds are toxic and deterrent to a broad range of phytophagous insects in oaks (Feeny 1970, Roslin and Salminen 2008, Moreira et al. 2018). We extracted phenolic compounds from 20 mg of dry leaf tissue with 1 ml of 70% methanol in an ultrasonic bath for 15 min, which was followed by centrifugation (Moreira et al. 2014). We then transferred the extracts to chromatographic vials. For phenolic quantification, we used Ultra-High-Performance Liquid-Chromatograph (UHPLC Nexera LC-30 AD; Shimadzu) equipped with a Nexera SIL-30AC injector and one SPD-M20A UV/VIS photodiode array detector. The compound separation was carried out on a Kinetex™ 2.6 μm C18 82–102 Å, LC Column 100 × 4.6 mm, protected with a C18 guard cartridge. The flow rate was 0.4 ml min^−1^ and the oven temperature was set at 25 °C. The mobile phase consisted of two solvents: water-formic acid (0.05%) (A) and acetonitrile-formic acid (0.05%) (B), starting with 5% B and using a gradient to obtain 10% B at 2 min, 20% B at 5 min, 30% B at 10, 40% B at 15, 60% B at 20, 80% B at 22 and 100% B at 23 min. The injection volume was 15 μl. For phenolic compound identification, we used an ultra-performance liquid chromatography coupled with electrospray ionization quadrupole (Thermo Dionex Ultimate 3000 LC) time-of-flight mass spectrometry (Bruker Compact™). We identified four groups of phenolic compounds: flavonoids, ellagitannins and gallic acid derivatives (‘hydrolysable tannins’ hereafter), proanthocyanidins (‘condensed tannins’ hereafter) and hydroxycinnamic acid precursors to lignins (‘lignins’ hereafter). We quantified flavonoids as rutin equivalents, condensed tannins as catechin equivalents, hydrolysable tannins as gallic acid equivalents and lignins as ferulic acid equivalents (Moreira et al. 2018, Galmán et al. 2019). We achieved the quantification of these phenolic compounds by external calibration using calibration curves at 0.25, 0.5, 1, 2 and 5 μg ml^−1^. We expressed phenolic compound concentrations in mg g^−1^ tissue on a dry weight basis.

The dataset was divided in two subsets: one at the leaf level used to fit photosynthetic light response curves and a second one at the tree level (with gas exchange measurements of different leaves aggregated per treatment) to explore the effects of species and induction treatment on leaf structural defenses, leaf chemical defenses and gas exchange.

### Statistical analyses

The leaf-level dataset was used to fit the Michaelis–Menten (MM: ${A}_{\mathrm{net}}=\frac{A_{\mathrm{sat},\mathrm{lc}}\,\bullet\, I\ }{K_m\,+\,I}-{R}_{\mathrm{dark},\mathrm{lc}}$), using the Global Optimization by differential evolution algorithm and minimizing the root sum of squares (Mullen et al. 2011, Fyllas et al. 2017). In the MM model, the net photosynthetic rate (*A*_net_) is a function of irradiance (*I*), light saturated photosynthetic rate (*A*_sat,lc_), half saturation coefficient (*K_m_*) and leaf dark respiration rate (*R*_dark,lc_), all measured in μmol m^−2^ s^−1^.

The tree-level dataset was used to analysis the average light saturated photosynthesis (*A*_sat_), the leaf dark respiration (*R*_dark_) and the leaf structural and defense traits using a linear multilevel model with species (two levels: *Q. coccifera* and *Q. pubescens*) and treatment (three levels: control, herbivory by *L. dispar* and mechanical damage) used as crossed random effects. In this model, individual trees were nested within species. For each trait, we initially fitted a random intercepts model for all terms (species/individual and treatment) and their species *×* treatment interaction using restricted maximum likelihood estimation. Thus, for each trait (*T*), the multilevel model is expressed by the equation:

(1) \begin{equation*} {T}_{\mathrm{sp},\mathrm{trt}}=\mu +{\alpha}_{\mathrm{sp}}+{\beta}_{\mathrm{ind}}+{\gamma}_{\mathrm{trt}}+{\left(\alpha \gamma \right)}_{\mathrm{sp},\mathrm{trt}}+{\varepsilon}_{\mathrm{sp},\mathrm{trt}} \end{equation*}

with *μ* as the grand mean of trait *T*, *α*_sp_ as the random species effect, *β*_ind_ as the random individual tree effect, *γ*_trt_ as the random treatment effect, (*αγ*)_sp,trt_ as the random species × treatment interaction term and *ε*_sp,trt_ as the residual variation. The random effects are assumed to follow a normal distribution with the variance components denoted as *σ*_sp_^2^, *σ*_ind_^2^  *σ*_trt_^2^, *σ*_sp,trt_^2^ for the species, individual, treatment and interaction term, respectively, with the residual variance denoted with *σ*^2^. In cases where the variance of a term was substantially (three orders of magnitude) lower that the variance of the other terms, the term was excluded, and a reduced model was refitted.

All data manipulation and graphs were made with the R ver. 4.0.3 (R Development Core Team 2020), the Tidyverse (Wickham et al. 2019) and the sjPlot (Lüdecke 2021) packages. The multilevel models were fitted with the lme4 (Bates et al. 2015) package.

## Results

### Effect of induction treatment on the shape of the light response curves

**Figure 2.**
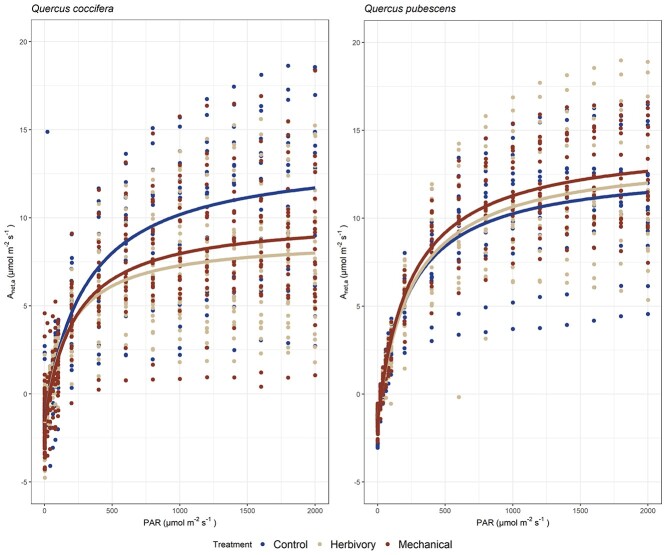
The mean light response curve (leaf-level dataset) per induction treatment (control, herbivory by *L. dispar*, mechanical damage) and oak species (*Q. coccifera* and *Q. pubescens*). Light response curves were developed by measuring net photosynthesis at different PAR levels and fitting a MM response curve afterward (see Materials and methods section).

The MM model was initially fitted (Figure 2) to light response data from the leaf-level dataset (81 from *Q. coccifera* and 48 from *Q. pubescens* leaves). For *Q. coccifera*, the inferred light saturated photosynthetic rate and the half saturation coefficient were greater in leaves from control branches compared with the other two induction treatments (*A*_sat.lc_: control: 15.07 ± 0.51, herbivory: 10.49 ± 0.21, mechanical damage: 11.64 ± 0.27 μmol m^−2^ s^−1^; *K_m_*: control: 301 ± 44, herbivory: 174 ± 16, mechanical damage: 220 ± 23 μmol quanta m^−2^ s^−1^). By contrast, the dark respiration rate did not differ between induction treatments (*R*_dark,lc_: control: 1.40 ± 0.25, herbivory: 1.64 ± 0.13, mechanical damage: 1.58 ± 0.16 μmol m^−2^ s^−1^).

For *Q. pubescens*, *A*_sat,lc_, *K_m_* and *R*_dark,lc_ did not differ between induction treatments (*A*_sat,lc_: control: 14.45 ± 0.30, herbivory: 15.11 ± 0.32, mechanical damage: 15.92 ± 0.21 μmol m^−2^ s^−1^; *K_m_*: control: 230 ± 21, herbivory: 254 ± 24, mechanical damage: 245 ± 14 μmol quanta m^−2^ s^−1^; *R*_dark_: control: 1.49 ± 0.17, herbivory: 1.40 ± 0.17, mechanical damage: 1.50 ± 0.12 μmol m^−2^ s^−1^).

The average light saturated photosynthesis (*A*_sat_) and the respiration measured after covering the leaf (*R*_dark_) were regressed against the *A*_sat,lc_ and the *R*_dark,lc_ parameters of the MM model, respectively. Both indicated a strong linear relationship (*A*_sat,lc_ = 0.11 + 1.04 ∙ *A*_sat_, *R*^2^ = 0.95; *R*_dark,lc_ = 0.04 + 0.94 ∙ *R*_dark_; *R*^2^ = 0.97), suggesting that using either of these two estimates was equivalent.

### Effect of induction treatment and oak species on photosynthesis and respiration

**Figure 3.**
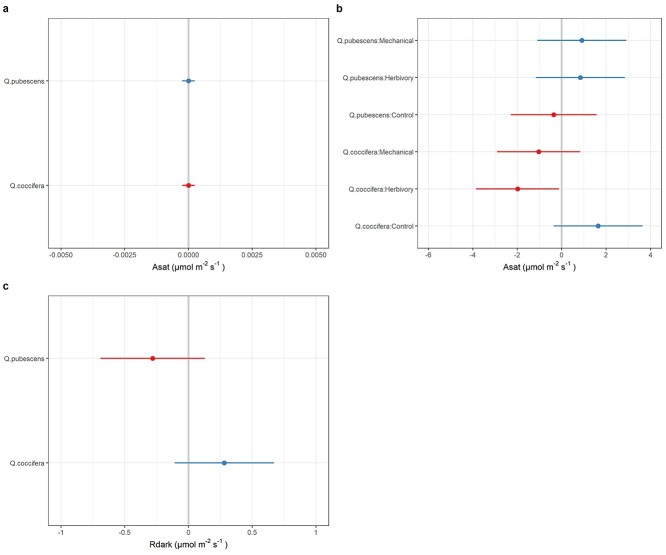
Effect of oak species (*Q. coccifera* and *Q. pubescens*) and oak species induction treatment (control, herbivory and mechanical damage) interaction on the light saturated photosynthesis (*A*_*sat*_) (a,b), and dark respiration (*R*_*dark*_) (c). Red colors indicate a negative random effect and blue colors indicate a positive random effect.

Average *A*_sat_ was similar between the two oak species, with *Q. coccifera* showing a relatively higher *R*_dark_ (Table 2). Most of the explained variation in *A*_sat_ was due to individual tree differences (*σ*_trt_/*σ*_tot_ = 3.01/16.65 = 18%) and species × treatment interactions (2.81/16.65 = 17%) (Table 3). The species random *A*_sat_ effects were similar between *Q. pubescens* and *Q. coccifera* (Figure 3a), with species × treatment effect suggesting that insect herbivory decreased *A*_sat_ for *Q. coccifera* but not for *Q. pubescens* (Figure 3b). A weaker negative effect of mechanical damage on *A*_sat_ was also found for *Q. coccifera*. The random effects of species identity for dark respiration were in general negative for *Q. pubescens* and positive for *Q. coccifera*, with 21% of the variation related to species identity and 36% related to within individual trees differences (Table 3, Figure 3c).

**Table 2:** Mean values (±1 SD) of the measured traits for each oak species and treatment.

Species Treatment	*Q. coccifera*			*Q. pubescens*		
	Control	Herbivory	Mechanical	Control	Herbivory	Mechanical
*E*_LA_	1.00 ± 0.00	0.87 ± 0.18	0.86 ± 0.21	1.00 ± 0.00	0.92 ± 0.15	0.88 ± 0.16
*L_A_*	3.46 ± 1.30	2.60 ± 1.09	2.48 ± 1.14	11.67 ± 3.52	10.25 ± 4.06	8.34 ± 2.55
*L*_th_	0.50 ± 0.06	0.50 ± 0.08	0.48 ± 0.07	0.51 ± 0.05	0.53 ± 0.11	0.52 ± 0.10
LDMC	0.62 ± 0.10	0.58 ± 0.07	0.61 ± 0.08	0.59 ± 0.08	0.59 ± 0.09	0.61 ± 0.08
LMA	194.5 ± 16.1	191.1 ± 21.4	181.2 ± 17.9	115.5 ± 15.8	123.2 ± 24.6	115.0 ± 13.8
Flavs	4.28 ± 0.71	4.05 ± 0.92	4.20 ± 0.74	6.17 ± 1.32	7.20 ± 2.08	7.68 ± 1.79
Lignins	0.06 ± 0.11	0.19 ± 0.12	0.24 ± 0.23	0.39 ± 0.38	0.63 ± 0.78	0.72 ± 0.64
CTannins	1.16 ± 0.52	0.92 ± 0.61	1.58 ± 0.82	3.33 ± 1.88	3.36 ± 2.34	2.58 ± 1.75
HTannins	1.42 ± 0.77	1.68 ± 0.83	1.67 ± 0.92	3.29 ± 0.81	3.27 ± 0.80	2.61 ± 0.72

**Table 3:** Summary of the crossed random effect models (Eq. (1)) for each trait. The intercept represents the grand mean value of each trait, while *σ*_sp_^2^, *σ*_trt_^2^, *σ*_sp,trt_^2^ are the species, treatment and interaction term variances, respectively, with the residual variance denoted with *σ*^2^. ICC is the intraclass correlation coefficient.

	*E*_LA_	*L_A_*	*L*_th_	LDMC	LMA	Flavs	Lignins	CTannins	HTannins
Intercept	0.919	6.469	0.504	0.599	153.366	5.586	0.366	2.156	2.330
Random effects									
*σ*^2^	0.243	4.970	0.006	0.007	357.232	1.472	0.147	1.677	0.689
*σ*_trt_^2^	0.004	0.708	0.000	0.000	11.160	0.000	0.011	0.000	0.000
*σ*_sp_^2^	0.000	25.994	0.000	0.000	2522.418	3.927	0.083	1.702	1.051
*σ*_sp,trt_^2^	0.000	0.452	0.000	0.000	5.187	0.197	0.000	0.074	0.033
Total variance	0.247	32.124	0.006	0.007	2895.997	5.596	0.241	3.453	1.774
ICC	0.017	0.845	0.052	0.017	0.877	0.737	0.390	0.514	0.611

### Effect of induction treatment and oak species on structural defenses

**Figure 4.**
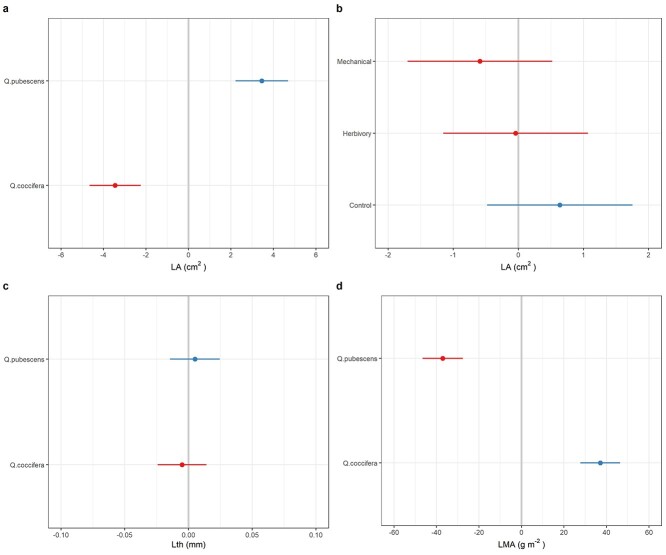
Effect of oak species (*Q. coccifera* and *Q. pubescens*) and induction treatment (control, herbivory and mechanical damage) on leaf structural defensive traits, as inferred from the random effect model: Leaf Area (a,b), Leaf thickness (c) and Leaf dry Mass per Area (d). Red colors indicate a negative random effect and blue colors indicate a positive random effect.

As expected, *E*_LA_ was lower in the treated leaves (19 and 24% lower in *Q. coccifera* leaves with insect herbivory and mechanical damage compared with control leaves, respectively, and 13 and 14% in *Q. pubescens*, Table 2). Leaves from the two oak species illustrated different *L_A_* and LMA, with the evergreen *Q. coccifera* having a lower *L_A_* and higher LMA compared with the semi-deciduous *Q. pubescens* (Table 2, Figure 4a and d). No differences were identified in terms of *L*_th_ and LDMC between species (Table 2, Figure 4c). The random effect analysis revealed little to no variation due to treatment (ranging from 0 to 2%) or due to species × treatment interaction (0% in all cases) for *L_A_*, *L*_th_, LDMC and LMA (Table 3). For example, only 0.578/29.472 = 2% of *L_A_* variation was due to induction treatment, with the negative effects of similar magnitude for the herbivory and mechanical damage induction treatments (Figure 4b).

### Effect of induction treatment and oak species on chemical defenses

**Figure 5.**
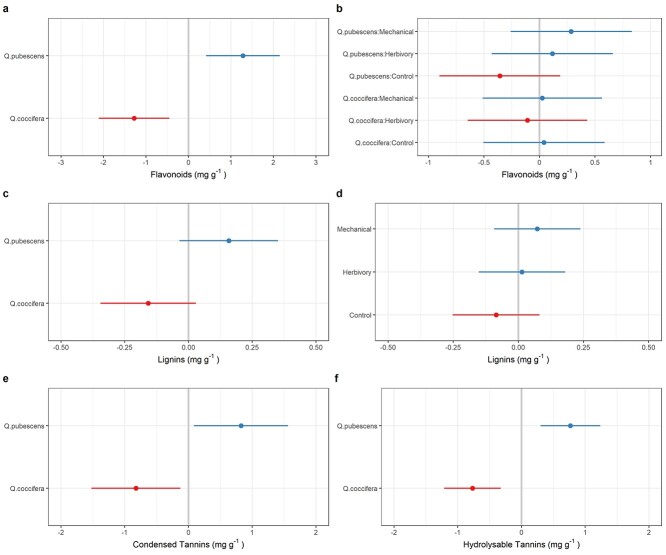
Effect of oak species (*Q. coccifera* and *Q. pubescens*), induction treatment (control, herbivory and mechanical damage) and their interaction on leaf chemical defensive traits, as inferred from the random effect model: Flavonoids (a,b), Lignins (c,d), Condensed Tannins (e) and Hydrolysable Tannins (f).

Oak species differed in all chemical defensive traits, with leaves of *Q. pubescens* having higher concentrations of chemical defenses (Table 2, Figure 5a, c, e and f). In particular, species identity accounted for 64, 22, 41 and 60% of flavonoids, lignins, condensed and hydrolysable tannins, respectively (Table 3). For flavonoids, the variation due to species × treatment interaction was 2.1% (Table 3), which translated to a 17 and 24% increased concentration for *Q. pubescens* in insect herbivory and mechanical damage (vs control), respectively, but not for *Q. coccifera* (Table 2, Figure 5b). Induction treatment accounted for 4.6% of lignins variation, with a positive random effect for both herbivory and mechanical damage (Figure 5d). Overall, lignins concentration was 237 and 316% higher in the herbivory and mechanical damage treatment for *Q. coccifera* and 60 and 83% higher in the herbivory and mechanical damage treatment for *Q. pubescens* (Table 2). Negligible fractions of variation were due to induction treatment for condensed and hydrolysable tannins.

## Discussion

### Photosynthesis-related traits

Our results showed that the two oak species did not strongly differ in their area-based photosynthesis capacity. Evergreen species are typically considered to exhibit a conservative resource-use strategy (relative to deciduous species), with lower photosynthetic and respiration rates (mass-based), which frequently results in reduced rates of resource allocation to growth and reproduction during the growing season (Givnish 2002, Lohbeck et al. 2015, Pérez-Ramos et al. 2015). By contrast, deciduous species exhibit an exploitative resource-use strategy (relative to evergreen species), with higher photosynthetic and respiration rates, traits commonly associated with a rapid resource capture, high relative growth rate and high investment in reproduction (Reich et al. 1998, Poorter and Garnier 2007, Fyllas et al. 2020). The similar area-based photosynthetic rates between the two oaks species observed in our study could be explained by the fact that *Q. pubescens* is a semi-deciduous species that retains its leaves ~7–8 months per year in our region (N. M. Fyllas, personal observation).

Although induction treatments and oak species did not significantly affect photosynthesis-related traits, we found evidence that these two factors exerted interactive effects on photosynthesis (Figure 3b). Specifically, insect herbivory (vs control) significantly decreased *A*_sat_ for *Q. coccifera* but not for *Q. pubescens*. One possibility to explain these findings would be the increased levels of some phenolic compounds after the induction treatments, which could result in a trade-off between resources used for photosynthesis versus chemical defense (Wasternack 2017).

### Leaf defensive traits

We found that oak species differ in their levels of defensive traits. For instance, *Q. pubescens* trees exhibit greater levels of chemical defenses (phenolic compounds) than *Q. coccifera* trees (Figure 5). By contrast, *Q. coccifera* trees exhibited greater structural defenses (smaller and tougher leaves, higher LMA) than *Q. pubescens* trees (Figure 4). Our results showed that investment in physical and chemical defenses by evergreen and deciduous oak species might evolve in the direction proposed by the Resource Availability Hypothesis (Coley et al. 1985, Endara and Coley 2011). Specifically, evergreen oak species, such as *Q. coccifera*, with theoretically lower growth rates and higher costs of tissue production and replacement should allocate more to physical defenses which are energetically more costly to produce and maintain than chemical defenses (Bonello et al. 2006, Moreira et al. 2014). Production of physical defenses involves processes of cell division and differentiation, whereas the production of chemical defenses usually involves only local changes in cell metabolism (Franceschi et al. 2005). Therefore, the former processes are slower, usually taking days or months, and are energetically more costly (depleting large amounts of carbohydrate reserves) in comparison with the synthesis of chemical defensive compounds (Franceschi et al. 2005). However, further studies comparing the costs in terms of energy of displaying several physical and chemical defenses in a wide array of oak species with contrasting leaf habit are needed to test this hypothesis.

We also found that both insect herbivory and mechanical damage increased lignin concentration for both species and flavonoid concentration for the semi-deciduous oak (Table 2, Figure 5). Inducibility (i.e., the ability to increase constitutive levels in response to damage) of these chemical defenses after natural or simulated herbivory has been shown to increase plant survival and provide an effective strategy for resistance against major insect herbivores (e.g., chewers, sap-feeders and miners) in several *Quercus* species (e.g., Mizumachi et al. 2012, Moreira et al. 2018, 2020, Galmán et al. 2021). Surprisingly, as previously reported for photosynthesis-related traits, induction of defenses by natural herbivory by *L. dispar* and mechanical damage produced similar quantitative results for flavonoids and lignins but not for tannins. Previous work has commonly found that plant-induced responses to herbivory are triggered not only by wounding but also by elicitors found in herbivore oral secretions or other body parts in the case of insects (including compounds involved in egg oviposition; Heil 2009, Hilker and Meiners 2010, Mithöfe and Boland 2012). However, a number of studies have contradicted the common view that mechanical wounding alone is not sufficient for the induction of herbivore-induced responses (e.g., Mithöfer et al. 2005, Moreira et al. 2012, Blackmon et al. 2016). For example, Mithöfer et al. (2005) found that leaf damage by the insect caterpillar *Spodoptera littoralis* and mechanical leaf wounding on lima bean (*Phaseolus lunatus*) plants induce a similar blend of volatile organic compounds. Similarly, Moreira et al. (2012) found that mechanical wounding and real phloem herbivory by *Hylobius abietis* on young maritime pine (*Pinus pinaster*) plants increased the concentration of resin and total phenolics by equivalent magnitudes. Further studies should address whether induced responses in other oak defensive (e.g., terpenes and alkaloids) or nutritional (e.g., nitrogen, phosphorus and carbon:nitrogen ratio) traits differ between real and simulated herbivory.

Finally, we found interactive effects of induction treatments and oak species in chemical defenses. In particular, insect herbivory and mechanical damage (vs control) significantly increased the concentration of flavonoids for *Q. pubescens* but not for *Q. coccifera*. Leaves of evergreen species, such as *Q. coccifera*, are available for a longer time and are constantly produced throughout the year (Orians and Solbrig 1977), which would in turn increase herbivores' chances of locating and completing their development on them. Under a scenario of higher herbivore pressure, it is possible that evergreen species (relative to deciduous species) will be inherently more predisposed to display other defensive strategies to protect (tougher) leaves or to quickly recover fitness from damage (e.g., tolerance mechanisms such as re-growth capacity or overcompensation in reproduction).

## Conclusion

Overall, this study shows that two congeneric oak species with contrasting leaf habit differ in their photosynthetic and defensive responses to insect herbivory. While the evergreen oak species followed a more conservative strategy (reduced photosynthesis, tougher leaves), the deciduous oak species followed a more acquisitive strategy (maintained photosynthesis, increased chemical defenses). Further studies should perform similar experimental set-ups including more species (even from different genera) and analyzing other secondary metabolites (e.g., terpenoids and alkaloids) or nutrients (e.g., nitrogen and phosphorus) to increase our understanding of the dynamics of plant–herbivore interactions under global change.

## Authors’ contributions

N.M.F. designed the research, analyzed the data and wrote the first version of the paper; D.C. carried out the data collection and made the photosynthesis and leaf traits measurements; D.N.A. contributed to species identification, larvae manipulation and contributed to the data analyses; and X.M. performed the chemical analyses and contributed to data analyses. All authors substantially contributed to revisions.
